# Persistence of norovirus in static and dynamic simulators of the human gastrointestinal tract

**DOI:** 10.3389/fmicb.2026.1750130

**Published:** 2026-03-19

**Authors:** Rosie Beaulieu, Éric Jubinville, Valérie Goulet-Beaulieu, Ismail Fliss, Julie Jean

**Affiliations:** Department of Food Science, Institut sur la nutrition et les aliments fonctionnels (INAF), Université Laval, Québec City, QC, Canada

**Keywords:** digestion, gastrointestinal tract, human norovirus, INFOGEST, murine norovirus, TIM-1

## Abstract

Human norovirus (HuNoV), transmitted via the fecal-oral route, is a major cause of gastroenteritis worldwide. However, its persistence during gastrointestinal digestion, particularly in the presence of high-risk food matrices, remains poorly understood. The aim of this study was to evaluate the persistence of HuNoV and murine norovirus 1 (MNV-1) during simulated gastrointestinal digestion using two complementary *in vitro* models: the standardized static INFOGEST protocol and the dynamic TIM-1 simulator. Viruses were suspended in water or mixed with puréed raspberries, strawberries, lettuce, or oysters. MNV-1 infectivity was quantified by plaque assays, complemented by PMAxx RT-qPCR, while HuNoV persistence was assessed exclusively by PMAxx-RT-qPCR as a proxy for potentially intact viral particles. Under static digestion conditions, MNV-1 titer decreased by approximately one log PFU/mL during the gastric phase in the presence of raspberry purée with a similar reduction observed for HuNoV RNA. In contrast, during dynamic digestion in TIM-1 system, both viruses persisted throughout all gastrointestinal compartments, regardless of the food matrix tested. These results highlight the persistence of noroviruses during gastrointestinal transit and reinforce the importance of preventive control measures for high-risk foods.

## 1 Introduction

Foodborne illnesses represent a major global public health burden, with an estimated 600 million cases annually worldwide, including approximately 125 million cases attributable to human norovirus (HuNoV) (World Health Organization, 2015). In Canada, HuNoV is responsible for more than 25% of the estimated four million annual foodborne illnesses (Thomas et al., 2013). HuNoV is transmitted via the fecal-oral route, either through direct person-to-person contact or through the consumption of contaminated food (Bosch et al., 2018).

Epidemiological investigations consistently identify specific food categories as high-risk vehicles for HuNoV transmission. According to joint FAO/WHO risk assessments, prepared foods are most frequently implicated, followed by frozen berries and bivalve shellfish (FAO/WHO, 2024). Leafy greens appear to be involved in the transmission of 9% of foodborne illnesses, with lettuce accounting for of the majority within this category (Yang and Scharff, 2024). Fresh and frozen berries have been linked to numerous outbreaks (Miotti et al., 2024) while bivalve shellfish can bioconcentrate HuNoV from contaminated waters with high prevalence reported at retail points of sale (McMenemy et al., 2023; Lowther et al., 2018). Together, these data illustrate the widespread prevalence of HuNoV across diverse food categories and support the selection of these matrices as representative high-risk vehicles for foodborne transmission in the present study.

Despite the public health relevance of HuNoV, experimental investigation of its behavior in the human gastrointestinal tract remains challenging. Although recent advances have enabled HuNoV replication in human intestinal enteroid models, these systems are complex, costly, and not always amenable to routine quantification of infectious virus. Consequently, murine norovirus 1 (MNV-1) and other cultivable caliciviruses remain widely used as surrogates in food virology research. MNV-1 can be readily propagated in cell culture, allowing direct measurement of infectivity by plaque assay, although it differs from HuNoV in receptor usage and host specificity (Richards, 2012). Nevertheless, MNV-1 has been extensively employed to estimate norovirus persistence and resistance under environmental and food-related stresses (Esseili et al., 2016).

A critical gap in current knowledge concerns the fate of noroviruses during gastrointestinal transit following ingestion of contaminated foods. Previous studies have examined norovirus or surrogate stability in simplified simulated digestive fluids or under non-standardized static conditions, often with short exposure times or without digestive enzymes and bile salts (Makison Booth and Frost, 2020; Joshi et al., 2015, 2017; Živković et al., 2021). Since the publication of the standardized INFOGEST protocol, studies have reported substantial persistence of norovirus surrogates during static digestion, primarily in water or selected food matrices (Brodkorb et al., 2019; Falcó et al., 2019; Lim et al., 2021; Riya and Malak, 2024). However, few studies have adhered strictly to the INFOGEST 2.0 conditions, and the influence of high-risk food matrices on viral persistence during gastrointestinal digestion remains incompletely understood.

Moreover, static digestion models do not capture key dynamic aspects of human digestion, such as progressive pH changes, gradual secretion of digestive fluids, peristaltic mixing, and compartmentalized transit through the small intestine. Dynamic gastrointestinal simulators, such as the TIM-1 system, allow a more physiologically realistic simulation of these processes and have been widely validated for the study of nutrient bio accessibility and microbial survival (Minekus et al., 1995; Verhoeckx et al., 2015). However, their application to the study of foodborne viruses remains limited.

The aim of this study was therefore to evaluate the persistence of HuNoV and MNV-1 during simulated gastrointestinal digestion in the presence of high-risk food matrices, using two complementary models: the standardized static INFOGEST protocol and the dynamic TIM-1 gastrointestinal simulator. By combining infectivity-based measurements for MNV-1 with RNA-based quantification of HuNoV following PMAxx pretreatment, this work provides an integrated assessment of norovirus persistence under physiologically relevant digestive conditions.

## 2 Materials and methods

### 2.1 Cell lines and preparation of viral stock suspensions

RAW 264.7 cells (ATCC^®^ TIB-71) were cultured in Dulbecco’s modified Eagle’s medium (DMEM) supplemented with 10% v/v fetal bovine serum and 1% v/v penicillin/streptomycin (all from Wisent, Inc., Quebec, Canada) and were frozen at −80 °C with 50 μL/mL dimethyl sulfoxide (Thermo Fisher Scientific, Waltham, Massachusetts, United States) until use. MNV-1 (ATCC^®^ VR-1937) was propagated in cell layers at MOI = 0.1 (multiplicity of infection). After 60 h of incubation at 37 °C under 5% CO_2_, the cells were lysed by freezing and thawing 3 times, and the lysate was centrifuged at 8,000 x g for 15 min (4 °C). The supernatant (containing the virus at a titer of 3.2 × 10^6^ pfu/mL) was aliquoted and frozen at −80 °C until use.

HuNoV GII.4 was isolated from an anonymized patient fecal sample (provided by Dr. Georges-L.-Dumont University Hospital Centre, Moncton, New Brunswick, Canada, IRB approval no. 2018-208 R-3/20-10-22022) as described previously (Trudel-Ferland et al., 2024). Briefly, the sample was diluted 1/10 in phosphate-buffered saline at pH 7.0 (PBS 1X) then centrifuged at 3,000 x g for 30 min (4 °C) and purified on an Amicon^®^ Ultra-4100 kDa column (UFC810024, MilliporeSigma, Burlington, MA, United States). The titer thus obtained was 4.67 × 10^7^ genome copies/μL, based on RT-qPCR (section 2.5).

The HuNoV GII.4 sample was genotyped at the Genomic Analysis Platform of the *Institut de Biologie intégrative et des Systèmes* (PAG-IBIS, Université Laval, Québec, QC, Canada) as described previously (Lévesque et al., 2021) using a BigDye™ Terminator v3.1 cycle sequencing kit (Applied Biosystems, Foster City, CA, United States) and analyzed using an ABI3500 genetic analyzer (Applied Biosystems, Thermo Fisher Scientific, United States) with 50-cm capillaries. The sequences were cleaned and aligned using Chromas v2.6.6 (Technelysium Pty Ltd., South Brisbane, Australia) and BioEdit v7.0.5.3 (Ibis Therapeutics, Carlsbad, CA, United States). Norovirus Automated Genotyping Tool v2.0 (Kroneman et al., 2011) was also used. The sequence was deposited in Genbank under accession number PV668482.

### 2.2 Foodstuffs

Distilled and sterilized water was used to wash the foods and as a control for foodstuff effects. Fresh strawberries, raspberries, and iceberg lettuce purchased at a local grocery store were washed 3 times and exposed to UV light for 15 min in a laminar flow hood at room temperature to inactivate natural microbiota. Live oysters (*Crassostrea virginica*) obtained from a local fishery (Les Cultures du Large, Îles-de-la-Madeleine, Québec, Canada) were washed and opened under sterile conditions. The whole oyster flesh was stored at −30 °C until use. All foods were pureed using a hand-held blender for 10 to 15 s to simulate mastication.

### 2.3 Digestion experiments under static conditions

The standardized INFOGEST 2.0 protocol (Brodkorb et al., 2019) was followed with the prescribed simulated salivary, gastric, and intestinal fluids. Digestions began with 5.0 ± 0.1 grams of water or food purée spiked with virus (MNV-1 at 6 log pfu/mL, HuNoV at 8 log genome copies per sample). Purée diluted with salivary fluid (1:1 wt/wt) was incubated at pH 7.0 ± 0.1 for 2 min. The oral bolus was diluted (1:1 wt/wt) with the gastric fluid and incubated for 2 h. The resulting chyme was mixed (1:1 wt/wt) with the intestinal fluid and incubated for another 2 h. The pH of each tube was adjusted before each phase and subsequently allowed to evolve according to the digestion phase conditions. Timing of each phase began after a five-minute stabilization. Each tube is considered an experimental unit, in accordance with the INFOGEST protocol. Tube contents were sampled at the end of the 2-min oral phase, 1 and 120 min into the gastric phase, and 1 and 120 min into the intestinal phase. Tubes containing HuNoV were sampled only at the end of each phase (at 2 min, 122 min, and 242 min) (Supplementary Figure 2). All samples were centrifuged immediately at 10,000 x g for 5 min (4 °C) and the supernatant was shaken, aliquoted, and frozen at −80 °C. To compare each digestion phase, the dilution factors considered were 10 (oral), 20 (gastric) and 40 (intestinal). Static digestions were performed for each food in triplicate, all on different days.

### 2.4 Dynamic digestion experiments

The persistence of MNV-1 infectivity and HuNoV RNA during passage through the human digestive tract was evaluated also using a dynamic simulator, namely the TIM-1 (TIM Company, Delft, The Netherlands) as recommended elsewhere (Minekus et al., 1995; Khalf et al., 2010). This model comprises four compartments, namely the stomach, the duodenum, the jejunum, and the ileum, all controlled by computer. The pH is adjusted in real time, while enzymes and electrolytes are pumped into the system. The temperature is maintained by water jackets surrounding each compartment. The whole system was maintained at 37 °C during all the digestion.

The flexible membrane and pumps provide peristaltic movement. The parameters were set to simulate 5-h digestion of a semi-solid meal by a healthy adult (Supplementary Table 1). The meal consisted of 30 grams of food mixed with 2 grams of artificial saliva (1 mg amylase (2,256 U/mg) in 45 mL of sterilized water) and brought to a final mass of 300 grams with sterile water. When the meal reached the stomach, 10 grams of gastric secretion at pH 2 were injected. Samples were taken from the gastric compartment at 0, 60 and 90 min and from the duodenum, jejunum, and ileum at 30, 150, and 300 min. Effluents and dialysates were sampled every 60 min (the different parts of the TIM-1 are represented in the Supplementary Figure 3).

The chyme remaining in each compartment at the end of the 5-h digestion was analyzed. The total recovery of virus was calculated using the sum of all ileal effluents. To estimate the persistence of infectious MNV-1 and intact HuNoV RNA (RNA detected after PMAxx pretreatment, used as a proxy for potentially intact viral particles) at each stage, the multiplicative coefficient was calculated using the percentage of the initial load (Table 1).


$$\text{Multiplicative coefficient}=\frac{1}{\left(\begin{array}{l} \%\text{of the load excluding}\;\\ \text{secretions}/100 \end{array}\right)}$$

**Table 1:** Multiplicative coefficient of enumeration relative to the initial sample in each compartment, versus time (Cordonnier et al., 2015).

Time	0	30	60	90	150	300
Stomach	1.00	-	1.06	1.11	–	–
Duodenum	–	1.95	–	–	1.44	4.75
Jejunum	–	8.91	–	–	1.36	1.80
Ileum	–	58.14	–	–	1.65	1.57

### 2.5 Measurement of MNV-1 persistence

Plaque assay was used to quantify the infectivity of MNV-1 during food digestion under static *in vitro* conditions. Plates (12-well) containing 8.5 × 10^5^ RAW 264.7 cells per well were incubated for 24 h at 37 °C under 5% CO_2_. The wells were then washed 2 times with 2 mL of PBS 1X. Digesta diluted serially 10-fold was spread on the cells, 300 μL in duplicate. Plates were incubated for 90 min then overlaid with 2.5 mL of minimal essential medium containing 10% fetal bovine serum, 2% L-glutamine, 10 mM HEPES, 0.3% sodium bicarbonate (all from Wisent Inc., Canada) and 0.8% SeaPlaque™ agarose (Lonza, Switzerland). Two days later, the plaques were fixed with 3.7% formaldehyde in saline (0.85% NaCl) for at least 5 h, and the wells were then coloured with 1% crystal violet to reveal the plaques.

The plaque assay was tested for sample cytotoxicity effects using digestions run in the absence of any food material (water only) and virus suspension. No overlay was applied. Inverted-phase microscopy images of cell layers were photographed.

Cell viability was tested quantitatively using an MTT-based cell proliferation kit I (Millipore Sigma, Canada). Corning 96-well plates contained 5 × 10^4^ cells and 100 μL of medium per well. After 24 h of incubation, 100 μL of tested solution was added, followed by 90 min of incubation. All liquid was then removed and 100 μL of DMEM with 10 μL of MTT (3-(4,5-dimethylthiazol-2-yl)-2,5-diphenyltetrazolium bromide) were added, followed by 2 h of incubation then 100 μL of solubilization solution for an additional 30 min of incubation. Absorbance at 570 nm was measured using a synergy H1 microplate reader (Biotek, Winooski, Vermont, United States).

### 2.6 Detection of intact HuNoV RNA

HuNoV was quantitated by RT-qPCR in samples pretreated with PMAxx (Biotium, Inc., Fremont, California, United States) as described by Lauzier et al. (2025). Briefly, the PMAxx stock solution (20 mM) was diluted at 1 mM in a 0.5% Triton solution. In the dark, PMAxx was added to 500 μL of every sample to reach a final concentration of 50 μM. The 1.5 mL tubes were immediately covered with aluminum foil to protect them from light. After 10-min agitation in the dark, samples were placed on ice and exposed to a halogen lamp for 15 min. Viral capsids were then lysed in 2 mL of NucliSens^®^ easyMAG™ lysis buffer (Biomérieux, Marcy-l’Étoileville, Auvergne-Rhône-Alpesregion, France). RNA was extracted using the semi-automated platform eGENE-UP (Biomérieux, Marcy-l’Étoile, France) following the manufacturer’s protocol and as previously described by Chatonnat et al. (2023).

To assess the potential impact of PCR inhibitors associated with food matrices, an additional column-based cleanup step (Zymo Research) was tested on selected samples prior to RT-qPCR analysis. Comparable genomes copy numbers were obtained with and without this cleanup step, suggesting that the observed reductions in RNA quantification and were therefore more likely related to sample recovery and matrix effects rather than amplification inhibition.

HuNoV RNA was quantified using an AB7500 real-time reverse transcriptase PCR system (RT-qPCR, Applied Biosytems, Thermo Fisher Scientific, Waltham, Massachusetts, United States) and data were compiled using Sequence Detection Software V1.5.1. A volume of 5 μL of RNA was added to 15 μL of reagent mixture comprising 10 μL of 2x iTaq universal probe reaction mixture, 0.625 μL of each primer and probe (417 nM), 0.5 μL of iScript advanced reverse transcriptase, and 2.63 μL of molecular-biology-grade water (VWR Avantor, Radnor, PA, United States) in a MicroAmp Optical 96-well reaction plate (Applied Biosystems, Waltham, Massachusetts, United States). The primers (Integrated DNA technologies (IDT), Inc., Coralville, Iowa, United States) and probes (Applied Biosystems, United States) are listed in Table 2. The plates were sealed with MicroAmp optical adhesive film (Applied Biosystems, United States). All samples were analyzed in duplicate.

**Table 2:** RT-qPCR primers and probes.

Virus	Identification	Sequence	Reference
HuNoV GII	QNFI2 (FW)	ATGTTCAGRTGGATGAGRTTCTCWGA	Loisy et al. (2005)
	COG2R (REV)	TCGACGCCATCTTCATTCACA	Kageyama et al. (2003)
	QNIFs (Probe)	6FAM-AGCACGTGGGAGGGCGATCGTAMRA	Loisy et al. (2005)
MNV-1	MNVF (FW)	TGCAAGCTCTACAACGAAGG	Hewitt et al. (2009)
	MNVR (RV)	CACAGAGGCCAATTGGTAAA	
	MNVP (Probe)	5Cy5-CCTTCCCGACCGATGGCATC-39IAbRQSp	

Quantification was performed using a standard curve generated from 10-fold serial dilutions (10^6^ to 10^1^) of pIDTSMART-AMP HuNoV GII (Integrated DNA technologies Inc., Coralville, Iowa, United States) prepared in 10 mM Tris with 0.1 mM EDTA. Each dilution was amplified in triplicate (Anonymous, 2017). The reverse transcription was run at 50 °C for 10 min, followed by polymerase activation and DNA denaturation at 95 °C for 3 min, and 45 cycles of thermal cycling at 95 °C for 15 s and 60 °C for 60 s.

### 2.7 Statistical analysis

All statistical analyses were performed using GraphPad prism version 10 (GraphPad Software Inc., California, United States). For static digestion experiments, the effect of digestion time was analyzed using one-way ANOVA followed by Tukey’s multiple comparison test. For dynamic digestion, the effects of time and food matrix on viral RNA recovery were analyzed using two-way ANOVA followed by Tukey’s *post hoc* test. Differences were considered statistically significant at *p* < 0.05.

For graphical representation, letters displayed above bars denote statistically homogeneous groups: values sharing at least one letter are not significantly different, whereas values that do not share any letter differ significantly (*p* ≤ 0.05). Consequently, value labeled “ab” do not differ significantly from values labeled “a” or “b.”

## 3 Results

### 3.1 Cytotoxicity of the digesta on the RAW 264.7 cells

No relevant cytotoxic effects of the digesta on RAW 264.7 cells were observed by microscopy (images not shown). Consistent with these observations, MTT viability assays confirmed that cell viability remained when samples were diluted at least 2-fold in the case of static digesta obtained at the 2 min, 3 min and 122 min points, at least 5-fold for digesta at the 123 min point, and at least 10-fold at the 242 min point (data not shown).

### 3.2 Persistence of infectious MNV-1 in foodstuffs during enzymatic digestion under static conditions (INFOGEST)

**Figure 1:**
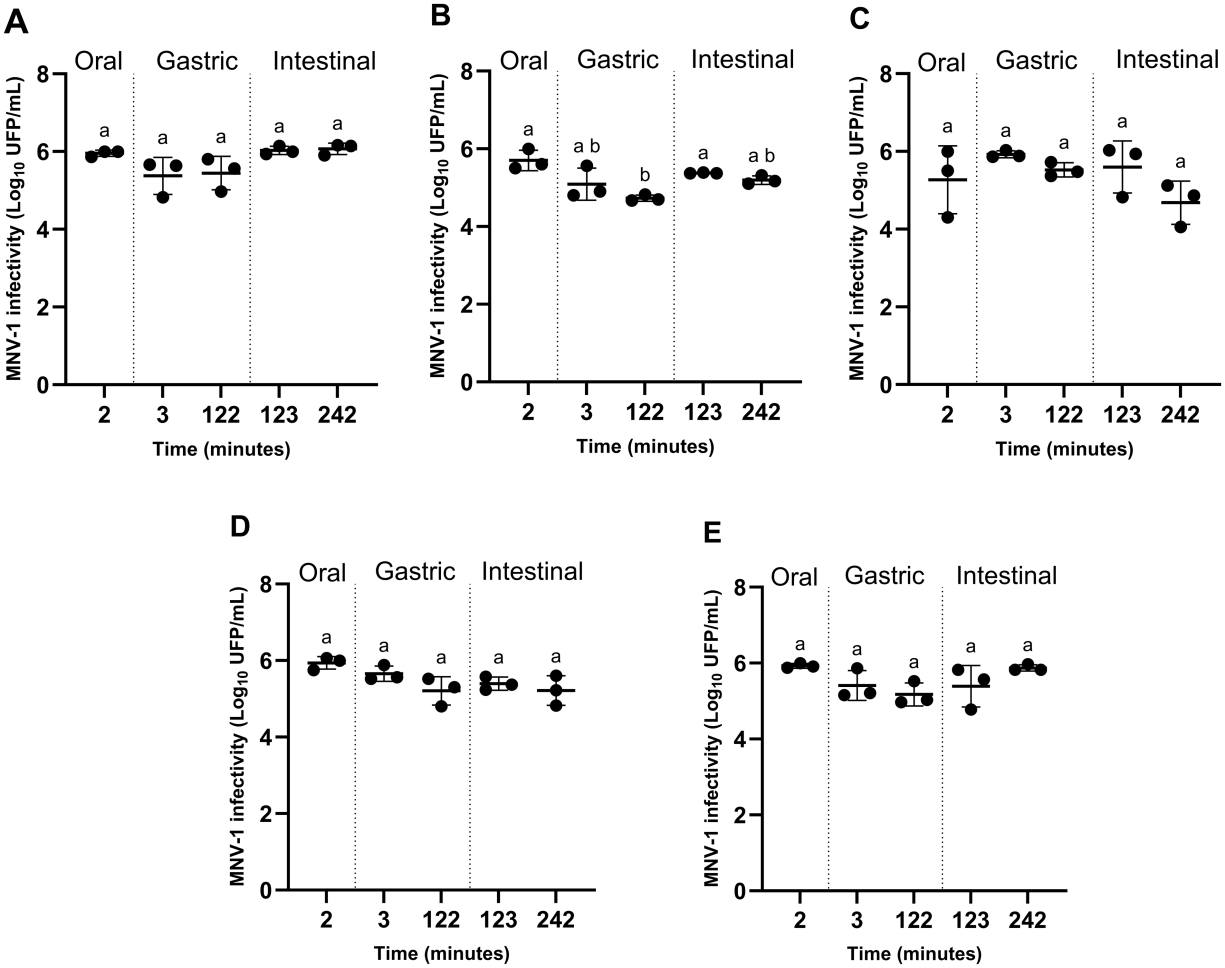
MNV-1 remaining infectious (based on plaque-forming assay) under static conditions representing the oral (2 min), gastric (3 and 122 min), and intestinal phases (123 and 242 min) of digestion in humans, with water **(A)**, with pureed raspberry, oyster, strawberry, or lettuce **(B–E)**. Values graphed are counts from each experiment and the mean ± standard deviation. Means with different letters differ significantly (*p* ≤ 0.05). All experiments were performed in triplicate.

Under static digestion conditions simulating the human gastrointestinal tract, MNV-1 infectivity remained largely stable throughout digestion for all tested food matrices (Figure 1). A reduction of approximately one log PFU/mL was observed during the gastric phase when MNV-1 was associated with raspberry purée (Figure 1B). No other significant decrease in infectivity relative to the initial oral condition was measured, including for virus suspended in water (Figures 1A,C–E).

MNV-1 persistence was also evaluated using RT-qPCR, with and without PMAxx pretreatment. Both approaches yielded highly comparable trends, with a maximal difference of ≤0.5 log₁₀ genome copies between conditions. For clarity and consistency, only PMAxx-RT-qPCR results are presented. Using this approach, a similar reduction in genome copies was observed during the gastric phase in the presence of raspberry purée, while viral RNA persisted through all other digestion phases and food matrices.

### 3.3 Persistence of HuNoV RNA in foodstuffs during enzymatic digestion under static conditions (INFOGEST)

**Figure 2:**
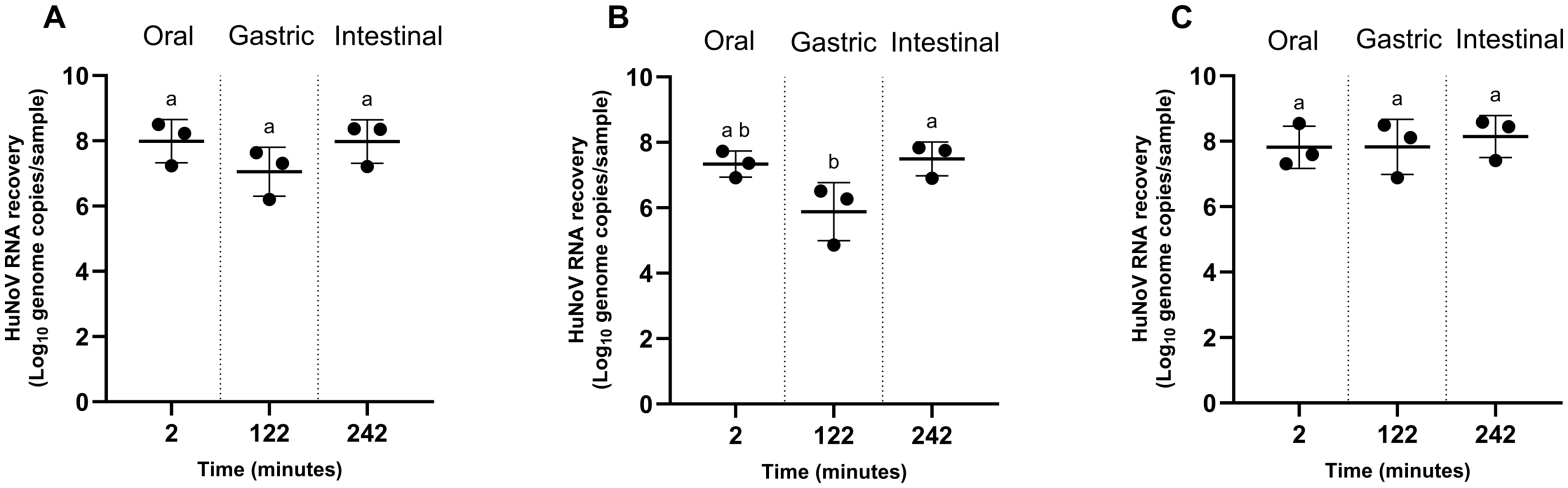
HuNoV RNA (based on PMAxx RT-qPCR) under static conditions representing the oral (2 min), gastric (122 min), and intestinal phases (242 min) of digestion in humans, with **(A)** water, **(B)** pureed raspberry, **(C)** pureed oyster. Values are counts from each experiment and the mean ± standard deviation. Means with different letters differ significantly (*p* ≤ 0.05). All experiments were performed in triplicate.

HuNoV RNA, quantified by PMAxx-RT-qPCR, remained detectable throughout the oral, gastric, and intestinal phases when suspended in water or mixed with pureed oyster (Figure 2). In contrast, when associated with pureed raspberry, a significant reduction of approximately 1.5 log of genomes copies per sample was observed during the gastric phase compared with the initial oral condition (Figure 2B). No additional significant decrease in HuNoV RNA levels was detected during the subsequent intestinal phase.

### 3.4 Persistence of MNV-1 RNA in a dynamic simulator of the human gastrointestinal tract (TIM-1)

**Figure 3:**
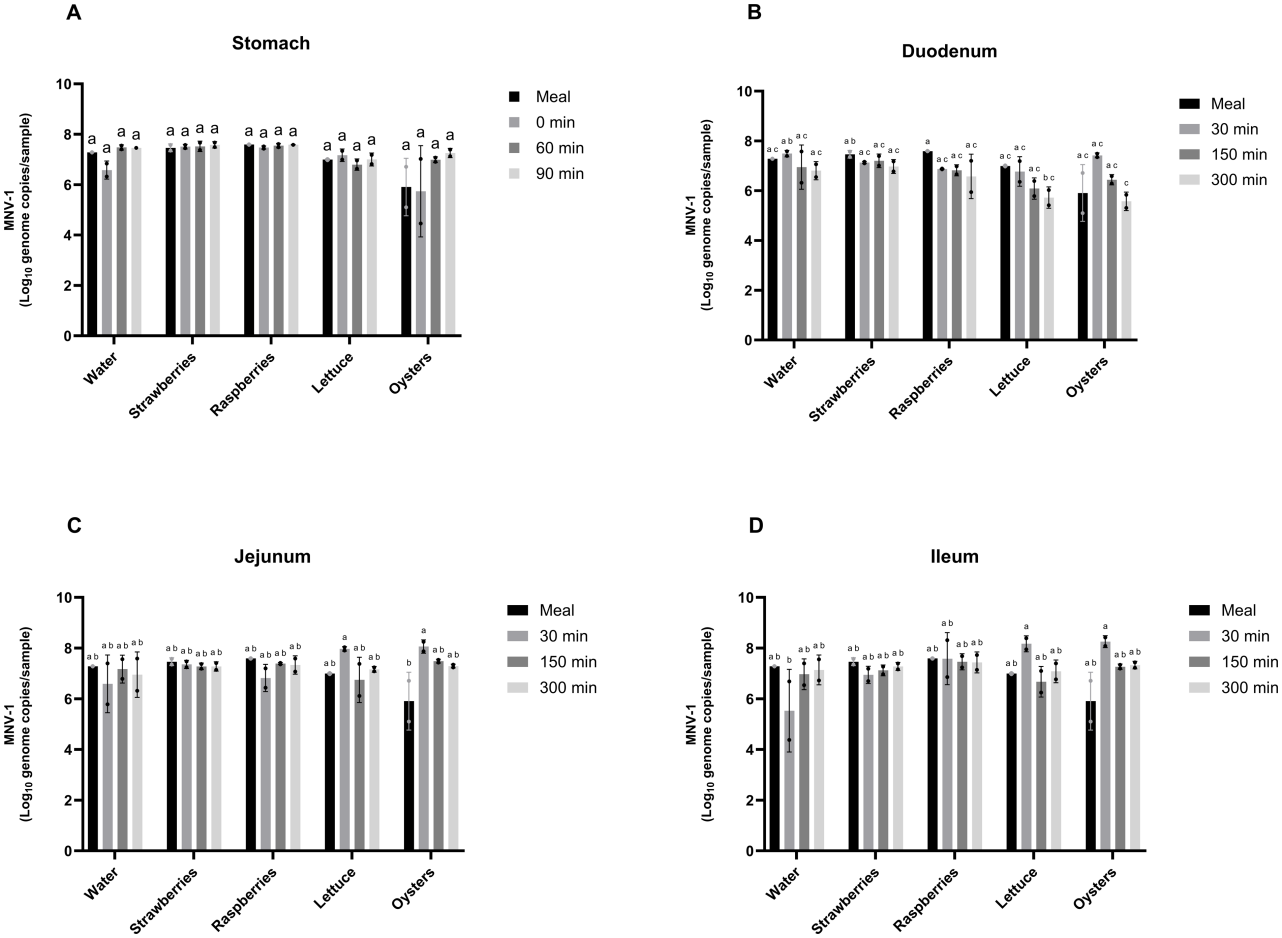
MNV-1 RNA copies (based on RT-qPCR) in the contents of the stomach **(A)**, duodenum **(B)**, jejunum **(C)**, and ileum **(D)** compartments of the TIM-1 gastrointestinal simulator during 5 h of digestion of water or pureed strawberry, raspberry, lettuce, or oyster. Data are presented as means ± standard deviation. Different letters indicate statistically significant differences (*p* ≤ 0.05) according to Tukey’s post-hoc test following a two-way ANOVA assessing the effects of food matrix and digestion time. All experiments were performed in duplicate.

MNV-1 RNA persistence during dynamic gastrointestinal digestion was assayed by RT-qPCR following PMAxx pretreatment in the four compartments of the TIM-1 system (Figure 3). Comparable trends were obtained when samples were analyzed without PMAxx pretreatment (data not shown).

In the stomach, no significant difference was observed between sampling times or food matrices (Figure 3A). In the duodenum, a few significant differences were observed between sampling time and food matrices; however, the magnitude of these variations generally remained below 2 log genome/copies (Figure 3B). MNV-1 RNA remained detectable across all food matrices in this compartment.

In the jejunum, MNV-1 RNA genome copies numbers remained relatively stable over time, although slightly lower levels were observed in samples associated with oyster purée compared with other matrices (Figure 3C). In the ileum, significant differences relative to the initial sampling time (30 min) were observed for water and oyster purée (Figure 3D). Specifically, an approximately 1.5 log reduction in RNA genome copies was observed for water relative to the initial meal, whereas an increase of approximately 2 log genome copies was observed relative for oyster purée. At later sampling times (150 and 300 min), no significant differences in MNV-1 RNA genome copy numbers were detected among food matrices.

### 3.5 Persistence of HuNoV RNA in a dynamic simulator of the human gastrointestinal tract (TIM-1)

**Figure 4:**
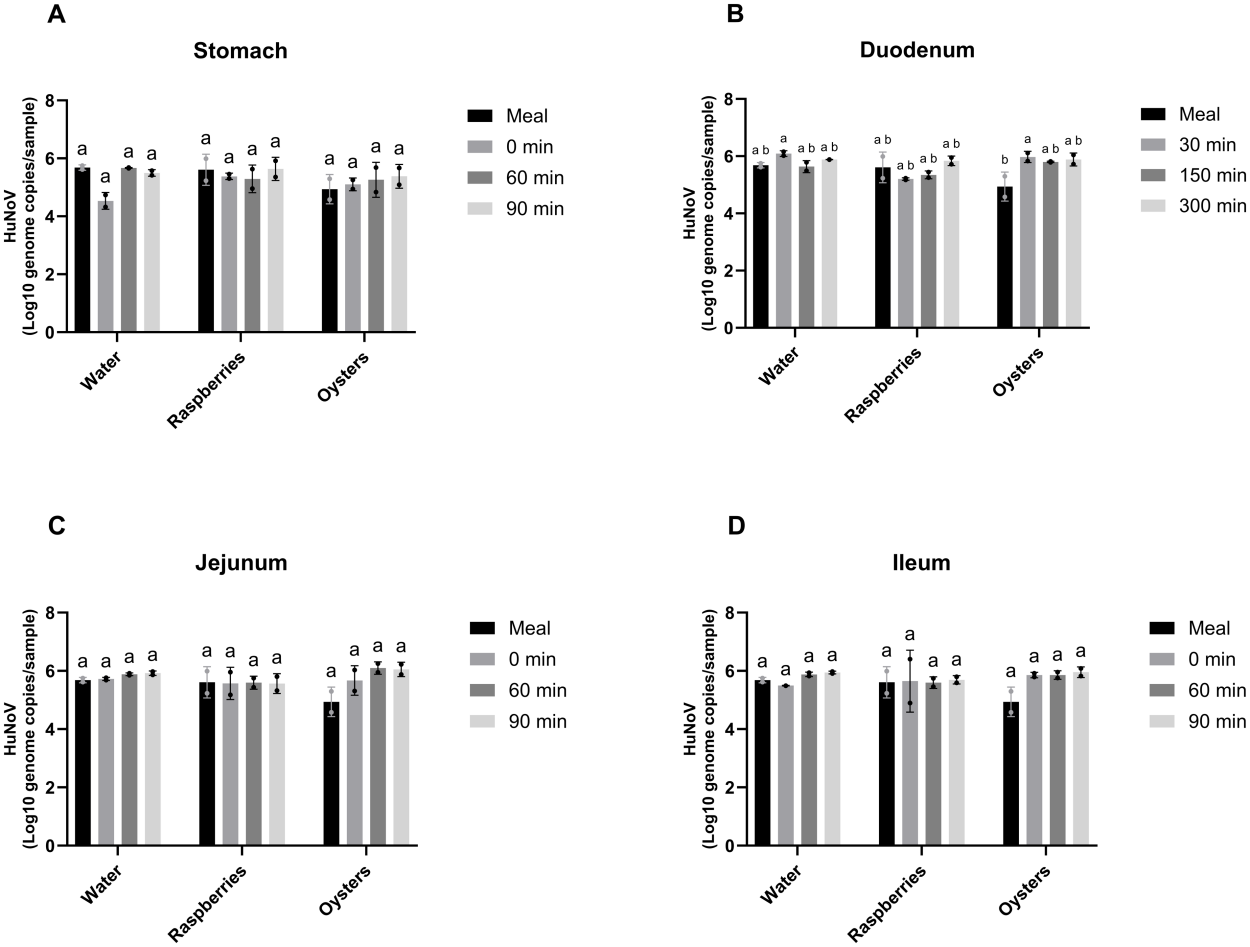
HuNoV RNA copies (based on RT-qPCR) in the contents of the stomach **(A)**, duodenum **(B)**, jejunum **(C)**, and ileum **(D)** compartments of the TIM-1 gastrointestinal simulator during 5 h of digestion of water or raspberry or oyster purée. Data are presented as means ± standard deviation. Different letters indicate statistically significant differences (*p* ≤ 0.05) according to Tukey’s post-hoc test following a two-way ANOVA assessing the effects of food matrix and digestion time. All experiments were performed in duplicate.

No significant changes in HuNoV genome copy numbers relative to the initial meal were observed in the stomach, jejunum or ileum compartments of the TIM-1 during the 5-h digestion, regardless of the food matrix tested (Figures 4A,C,D). In the duodenum, HuNoV RNA genome copy numbers measured in oyster purée at 30 min were significantly higher than the initial value measured in the meal, corresponding to with an increase of approximately 1 log of RNA genome copies (Figure 4B). This was the only statistically significant difference observed relative to the initial meal across compartments and food matrices.

### 3.6 Quantification of virus in the outflows from the TIM-1

The majority of MNV-1 and HuNoV RNA introduced into the TIM-1 system was recovered in the ileal effluent collected at the end of the digestion, based on RT-qPCR detection of the viral RNA (Table 3).

**Table 3:** Virus in the chyme flowing from the TIM-1 gastrointestinal simulator at the end of the digestion.

Log_10_ genome copies injected	Sample	Log_10_ viral genome copies recovered
MNV-1 7.58 ± 0.07	Water	7.56 ± 0.50
	Strawberry	7.60 ± 0.14
	Raspberry	7.80 ± 0.07
	Lettuce	7.53 ± 0.00
	Oyster	7.79 ± 0.02
HuNoV GII.4 6.17 ± 0.03	Water	6.30 ± 0.02
	Raspberry	6.24 ± 0.40
	Oyster	6.49 ± 0.24

Counts are scaled to the entire volume injected and expressed as mean ± standard deviation (*n* = 2).

A total of 50 dialysate samples were analyzed for the presence of MNV-1 RNA and 30 for HuNoV RNA. MNV-1 RNA was detected in 15 out of 50 dialysates, with quantities ranging from 33 to 5,300 RNA genome copies per sample (Table 4). In contrast, HuNoV RNA was not detected in any of the dialysate samples analyzed.

**Table 4:** MNV-1 and HuNoV RNA detected in dialysates from the TIM-1 gastrointestinal simulator.

Virus	Number of positive dialysates	Genome copies/sample	C_q_ range
MNV-1	15/50	505.8 ± 1,335 (min: 33 max: 5300)	31.51–39.15
HuNoV	0/30	–	–

## 4 Discussion

In this study we investigated the persistence of HuNoV and MNV-1 under conditions characterizing the human digestive system, using foodstuffs frequently involved in the transmission of norovirus-caused gastroenteritis. MNV-1 is frequently used as a proxy for HuNoV in studies of norovirus infectivity. The two protocols used to simulate human gastrointestinal tract conditions were INFOGEST (a static model) and the TIM-1, an apparatus that mimics the sequence of enzymatic actions and pH encountered by ingested food as it passes through the stomach, the duodenum, the jejunum, and the ileum. Static digestion is a reproducible, simplified and relatively low-cost method for our purposes (Brodkorb et al., 2019). However, it does not replicate the dynamics that occur during real digestion, such as the gradual addition of gastric fluids, the progressive gastric emptying, or the segmentation of the intestinal phase into its three distinct stages (Brodkorb et al., 2019). Because HuNoV was quantified exclusively by PMAxx-RT-qPCR, persistence is discussed in terms of RNA genome copies and potentially intact viral particles, and not as a direct measure of infectivity.

### 4.1 Impact of pH on noroviruses

In the present study, neither HuNoV RNA nor MNV-1 infectivity showed substantial loss during exposure to simulated gastric and intestinal conditions in either static or dynamic digestion models, regardless of the food matrix tested. These findings indicate that exposure to gastrointestinal pH conditions alone is insufficient to markedly reduce norovirus persistence.

To successfully establish infection following ingestion, enteric viruses must withstand the acidic environment of the stomach, digestive enzymes, and mechanical stresses associated with gastrointestinal transit. Consistent with our observations, previous studies have reported high stability of HuNoV and norovirus surrogates under acidic conditions. Early human challenge studies demonstrated that HuNoV retained infectivity following prolonged exposure to low pH (Kapikian et al., 1972) while more recent *in vitro* studies have shown sustained detectability of HuNoV RNA in simulated gastric fluids at pH values comparable to those used in the present work (Tung-Thompson et al., 2015). Furthermore, noroviruses can replicate in human intestinal enteroids derived from the duodenum, jejunum, and ileum, but not in colonic enteroids. The ability of GII.4 to reach the small intestine and replicate in these cells underscores that the virus can remain infectious as it transits through the gastrointestinal tract (Ettayebi et al., 2024).

Together, these findings support the conclusion that gastrointestinal pH conditions, in isolation, do not constitute a major barrier to norovirus persistence during digestion.

### 4.2 Persistence of noroviruses in food matrices under gastrointestinal conditions

In the present study, the presence of food matrices did not markedly enhance viral inactivation during either static or dynamic gastrointestinal digestion. Except for raspberry purée under static gastric conditions, both MNV-1 infectivity and HuNoV RNA remained largely stable throughout digestion when associated with the tested food matrices. These observations suggest that, rather than promoting viral inactivation, food matrices may contribute to the persistence of noroviruses during gastrointestinal transit.

Several mechanisms may account for the apparent protective effect of food matrices observed in this study. Food components can buffer acidic conditions, limit exposure to digestive enzymes, and physically entrap viral particles within the food matrix. Consistent with this interpretation, previous studies have shown that berries and other plant-based foods can adsorb noroviruses through electrostatic interactions, thereby reducing viral exposure to environmental stressors (Le Guyader and Atmar, 2008; Pitol et al., 2017; Bozkurt et al., 2021). Such interactions may also influence viral recovery following digestion, particularly for matrices that are not fully solubilized during gastrointestinal processing.

Shellfish represent another high-risk matrix in which norovirus persistence has been widely documented. Bivalve mollusks can bioaccumulate noroviruses from contaminated waters through specific ligand-mediated interactions, and these associations may persist during digestion. In the present study, both MNV-1 RNA and HuNoV RNA remained detectable throughout dynamic digestion when associated with oyster purée, supporting the notion that shellfish matrices can preserve viral material during gastrointestinal transit.

It is important to note that viral persistence in this study was primarily assessed using RNA-based methods, particularly for HuNoV. Consequently, the observed stability reflects the persistence of viral RNA and potentially intact viral particles, rather than direct measurements of infectivity. Nonetheless, the limited reduction observed across most food matrices underscores the robustness of noroviruses and highlights the potential of contaminated foods to serve as efficient vehicles for viral exposure.

### 4.3 Contribution of static and dynamic digestion models to the assessment of norovirus persistence

The combined use of a standardized static digestion model (INFOGEST) and a dynamic gastrointestinal simulator (TIM-1) provided complementary insights into norovirus persistence during simulated human digestion. While static digestion offers a reproducible and cost-effective approach to assess viral stability under well-defined conditions, it does not account for the dynamic processes that characterize gastrointestinal transit, such as gradual pH changes, progressive secretion of digestive fluids, peristaltic mixing, and compartmentalized passage through the small intestine.

By incorporating these physiological features, the TIM-1 system allowed a more realistic evaluation of viral fate during digestion. The consistency observed between static and dynamic models, particularly the limited reductions in viral RNA or infectivity across most conditions, reinforces the robustness of noroviruses under gastrointestinal stresses. Importantly, the dynamic model enabled compartment-specific analysis, revealing localized variations in viral RNA levels that would not be captured under static conditions alone.

Together, these findings highlight the value of integrating static and dynamic digestion models to better approximate human exposure scenarios and to improve the assessment of foodborne virus persistence under physiologically relevant conditions.

## 5 Conclusion

This study investigated the persistence of noroviruses under simulated human gastrointestinal conditions using complementary static and dynamic digestion models. Under static digestion, MNV-1 remained infectious when suspended in water or mixed with purées of high-risk food matrices, except for raspberry purée, which induced an approximately 1-log reduction during the gastric phase. Similarly, HuNoV RNA levels were largely maintained throughout digestion, with a comparable reduction observed during the gastric phase in the presence of raspberry purée.

During dynamic digestion using the TIM-1 system, both MNV-1 RNA and HuNoV RNA remained detectable across gastrointestinal compartments and food matrices, with only limited and compartment-specific variations. Although HuNoV infectivity was not directly assessed, the persistence of viral RNA following PMAxx pretreatment suggests that potentially intact viral particles can withstand gastrointestinal transit under the tested conditions.

Overall, these findings indicate that gastrointestinal digestion alone is insufficient to substantially reduce norovirus persistence, particularly when viruses are associated with high-risk food matrices. The integration of static and dynamic digestion models provides a more comprehensive framework to assess foodborne virus persistence and reinforces the importance of preventive control measures to limit norovirus contamination in the food supply.

## Data Availability

The datasets presented in this study can be found in online repositories. The names of the repository/repositories and accession number(s) can be found at: https://www.ncbi.nlm.nih.gov/nuccore/PV668482.
