# Supplementary material for: Persistence of norovirus in static and dynamic simulators of the human gastrointestinal tract
Source: Front Microbiol. 2026 Mar 19;17:1750130. doi: 10.3389/fmicb.2026.1750130 (PMC13043994; doi:10.3389/fmicb.2026.1750130)
Supplement: Supplementary file 1 [file Data_Sheet_1.docx]

**Supplementary data**

Supplementary Table 1: TIM-1 program used in this study

|  |  | **0** | **30** | **60** | **90** | **120** | **150** | **180** | **240** | **300** |
| --- | --- | --- | --- | --- | --- | --- | --- | --- | --- | --- |
| **Stomach** | **Volume (mL)** | 310 | 274 | 194 | 116 | 61 | 29 | 14 | 6 | 4 |
|  | **%OI*** | 100 | 86 | 59.15 | 33.64 | 16.06 | 6.51 | 2.17 | 0.17 | 0.01 |
|  | **%**** | 99.92 | 97.44 | 94.37 | 89.82 | 82.17 | 68.73 | 46.88 | 8.65 | 0.45 |
|  | **output** |  | 44.14 | 130.96 | 216.67 | 279.65 | 318.41 | 341.17 | 364.09 | 381.25 |
|  | **mL/sec** | 0.25 | 0.25 | 0.25 | 0.25 | 0.25 | 0.25 | 0.25 | 0.25 | 0.25 |
|  | **pH** | 5.5 | 3.5 | 1.8 | 1.8 | 1.7 | 1.7 | 1.7 | 1.7 | 1.7 |
| **Duodenum** | **Volume (mL)** | 55 | 55 | 55 | 55 | 55 | 55 | 55 | 55 | 55 |
|  | **%OI** |  | 9.11 | 14.39 | 14.82 | 13.89 | 12.35 | 10.36 | 6.54 | 3.73 |
|  | **%** |  | 51.36 | 81.13 | 83.54 | 78.31 | 69.62 | 58.38 | 36.88 | 21.04 |
|  | **output** | 0.25 | 51.89 | 146.21 | 239.42 | 309.9 | 356.16 | 386.67 | 424.34 | 457 |
|  | **mL/sec** | 0.25 | 0.25 | 0.25 | 0.25 | 0.25 | 0.25 | 0.25 | 0.25 | 0.25 |
|  | **pH** | 6 | 6 | 6 | 6 | 6 | 6 | 6 | 6 | 6 |
| **Jejunum** | **Volume (mL)** | 115 | 115 | 115 | 115 | 115 | 115 | 115 | 115 | 115 |
|  | **%OI** |  | 4.16 | 16.82 | 24.62 | 27.11 | 27.23 | 26.43 | 23.95 | 20.59 |
|  | **%** |  | 11.22 | 45.33 | 66.37 | 73.07 | 73.41 | 71.24 | 64.55 | 55.5 |
|  | **output** |  | 51.64 | 145.96 | 239.17 | 309.65 | 355.91 | 386.42 | 424.09 | 456.75 |
|  | **Abs** | 0.25 | 0.25 | 0.25 | 0.25 | 0.25 | 0.25 | 0.25 | 0.25 | 0.25 |
|  | **Dialysed** | 0 | 300 | 600 | 900 | 1200 | 1500 | 1810 | 2400 | 3020 |
|  | **pH** | 6.5 | 6.5 | 6.5 | 6.5 | 6.5 | 6.5 | 6.5 | 6.5 | 6.5 |
| **Ileum** | **Volume (mL)** | 115 | 115 | 115 | 115 | 115 | 115 | 115 | 115 | 115 |
|  | **%OI** |  | 0.64 | 6.79 | 15.07 | 20.15 | 22.5 | 23.52 | 24 | 23.56 |
|  | **%** |  | 1.72 | 18.31 | 40.61 | 54.32 | 60.66 | 63.39 | 64.7 | 63.51 |
|  | **Output** |  | 51.64 | 145.96 | 239.17 | 309.65 | 355.91 | 386.42 | 424.09 | 456.75 |
|  | **Dialysed** | 0 | 300 | 600 | 900 | 1200 | 1500 | 1810 | 2400 | 3020 |
|  | **pH** | 7.2 | 7.2 | 7.2 | 7.2 | 7.2 | 7.2 | 7.2 | 7.2 | 7.2 |

***%OI: Meal distribution throughout the entire system**

****%: Percentage of the meal excluding secretions**


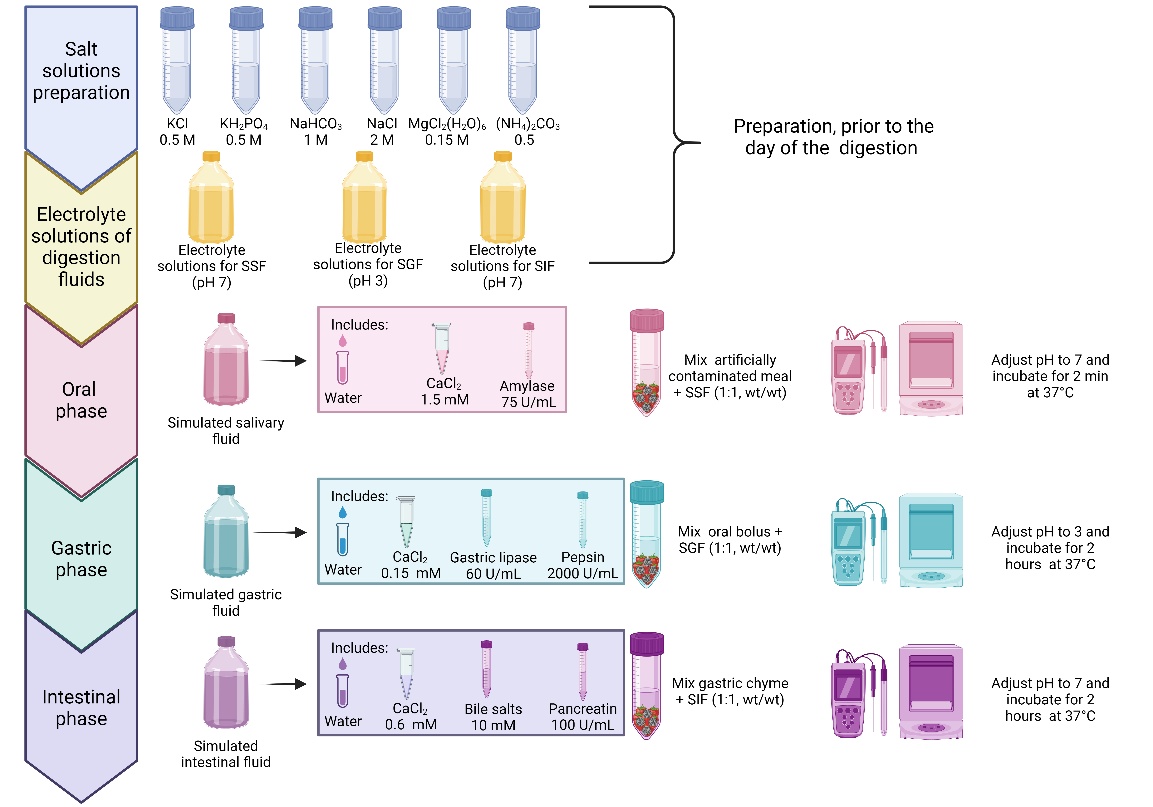


*Samples were taken after this step.

**Samples were taken after the pH was adjusted to 3.

***Samples were taken after the 2 hours incubation.

****Samples were taken after the pH was adjusted to 7.

*****Samples were taken after the 2 hours incubation.

Supplementary figure 2 : Infographic representation of the INFOGEST 2.0 protocol and the sampling related to this experiment.


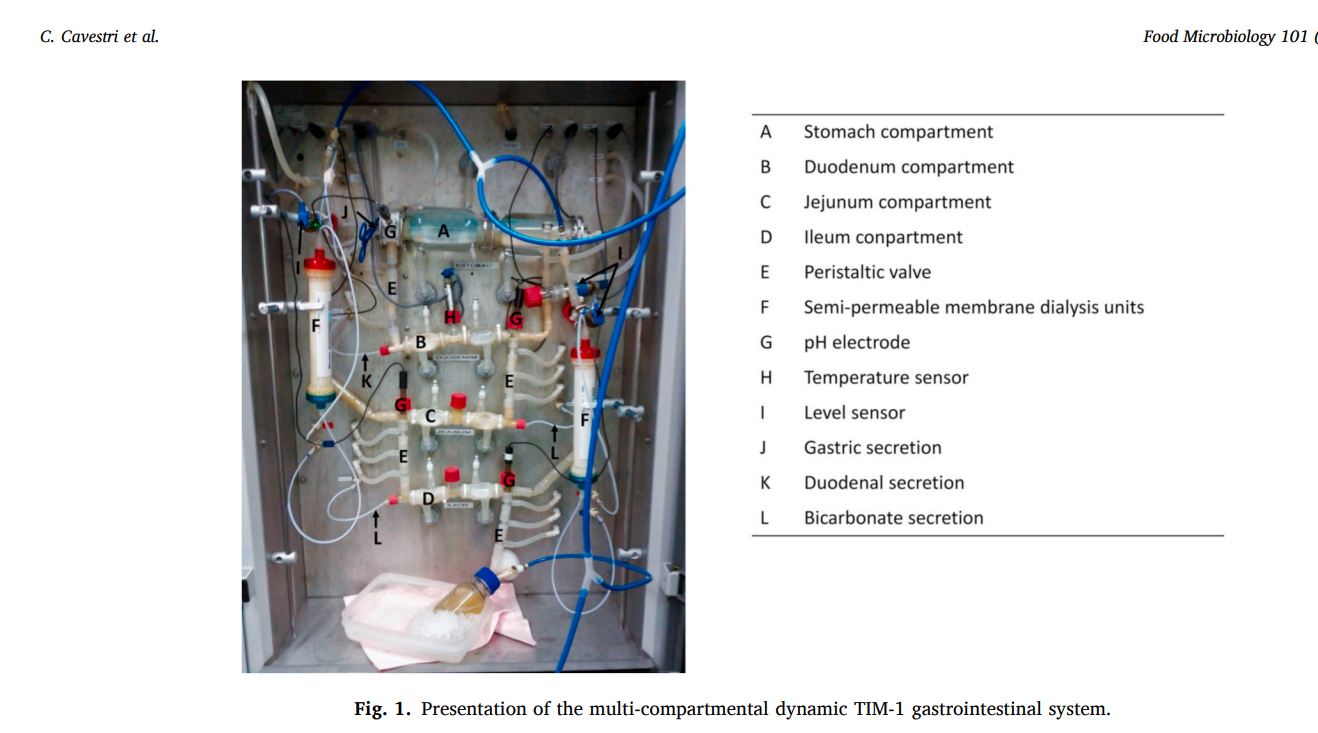


Supplementary figure 3: Figure reproduced from Cavestri et al., showing the structural organization and functional operation of the TIM-1 model. The fraction of the meal that passes through the semi-permeable membrane dialysis (F) units goes into a bottle under the TIM-1, it is the dialysates. The fraction of the meal that passes through all the digestive system and reaches the bottle at the end represent the effluents, the chyme that would normally reach the large intestine.
